# Novel cell culture system for monitoring cells during continuous and variable negative‐pressure wound therapy

**DOI:** 10.1111/srt.13262

**Published:** 2022-12-25

**Authors:** Toshifumi Yamashiro, Toshihiro Kushibiki, Yoshine Mayumi, Masato Tsuchiya, Miya Ishihara, Ryuichi Azuma

**Affiliations:** ^1^ Department of Plastic and Reconstructive Surgery National Defense Medical College Tokorozawa Saitama Japan; ^2^ Department of Medical Engineering National Defense Medical College Tokorozawa Saitama Japan

**Keywords:** epithelial‐to‐mesenchymal transition, intermittent negative‐pressure wound therapy, keratinocyte, microdeformational wound therapy, negative‐pressure incubator, topical negative pressure, vacuum‐assisted closure, wound healing

## Abstract

**Background:**

Although the clinical efficacy of negative‐pressure wound therapy (NPWT) is well known, many of its molecular biological mechanisms remain unresolved, mainly due to the difficulty and paucity of relevant in vitro studies. We attempted to develop an in vitro cell culture system capable of real‐time monitoring of cells during NPWT treatment.

**Materials and methods:**

A novel negative‐pressure cell culture system was developed by combining an inverted microscope, a stage‐top incubator, a sealed metal chamber for cell culture, and an NPWT treatment device. Human keratinocytes, PSVK‐1, were divided into ambient pressure (AP), continuous negative‐pressure (NPc), and intermittent negative‐pressure (NPi) groups and cultured for 24 h with scratch assay using our real‐time monitoring system and device. Pressure inside the device, medium evaporation rate, and the residual wound area were compared across the groups.

**Results:**

Pressure in the device was maintained at almost the same value as set in all groups. Medium evaporation rate was significantly higher in the NPi group than in the other two groups; however, it had negligible effect on cell culture. Residual wound area after 9 h evaluated by the scratch assay was significantly smaller in the NPc and NPi groups than in the AP group.

**Conclusion:**

We developed a negative‐pressure cell culture device that enables negative‐pressure cell culture under conditions similar to those used in clinical practice and is able to monitor cells under NPWT. Further experiments using this device would provide high‐quality molecular biological evidence for NPWT.

## 1 INTRODUCTION

Since its first report by Morykwas et al. in 1997,^1^ negative‐pressure wound therapy (NPWT) has become an integral part of clinical wound care. Its efficacy has expanded to include difficult‐to‐treat lesions, such as diabetic foot ulcers^2^ or burns,^3^ and with the evolution of advanced devices, its application in infected wounds,^4^ which was initially considered unsafe, has also been confirmed effective. In recent years, variable NPWT, including intermittent NPWT, in which high negative pressure and low or no negative pressure are periodically repeated, has been attracting attention; it has been shown to be more effective in terms of therapeutic effects, such as angiogenesis, than continuous negative pressure, in which a constant negative pressure is continuously applied.^5^, ^6^ Although the effectiveness of NPWT in clinical practice is widely recognized, basic in vitro studies using cells are still few in number, and the mechanism underlying NPWT effects remains largely unknown. An important reason for the paucity of basic research is that NPWT is established by a complex interplay of therapeutic mechanisms that are difficult to reproduce in vitro. These mechanisms include the drainage of exudate and bacteria by negative pressure, coarse contraction effect on the wound surface (macrodeformation), cellular response to negative pressure (microdeformation), and the effect of dressings on moisture and heat retention and as barriers from the external environment.^7^, ^8^, ^9^ As it is difficult to include all of these parameters in a single experimental system in vitro, studies focusing on individual mechanisms are urgently required.

There have been several reports on cellular responses under negative pressure, including some that created special negative‐pressure culture devices,^10^, ^11^, ^12^ and others that used simple measures to bring culture flasks to negative pressure.^13^, ^14^ All of them involve interruption of the treatment (i.e., moving the cells to ambient pressure) when observing live cells and do not allow a continuous observation of cultured cells under negative pressure. Furthermore, there are limited laboratories in which this type of experiment can be performed. In order to overcome these problems, we attempted to develop a negative‐pressure cell culture system, that is, simple and reproducible. By combining a sealed chamber for cell culture with a clinically used negative‐pressure, therapy device, we succeeded in reproducing a negative‐pressure environment on a microscope stage without the need for extensive equipment, and in real‐time monitoring of cultured cells in a monolayer under negative pressure. Results of several experiments using this device are reported herein.

## 2 MATERIALS AND METHODS

### 2.1 Equipment for culturing cells on a microscope stage

**FIGURE 1:**
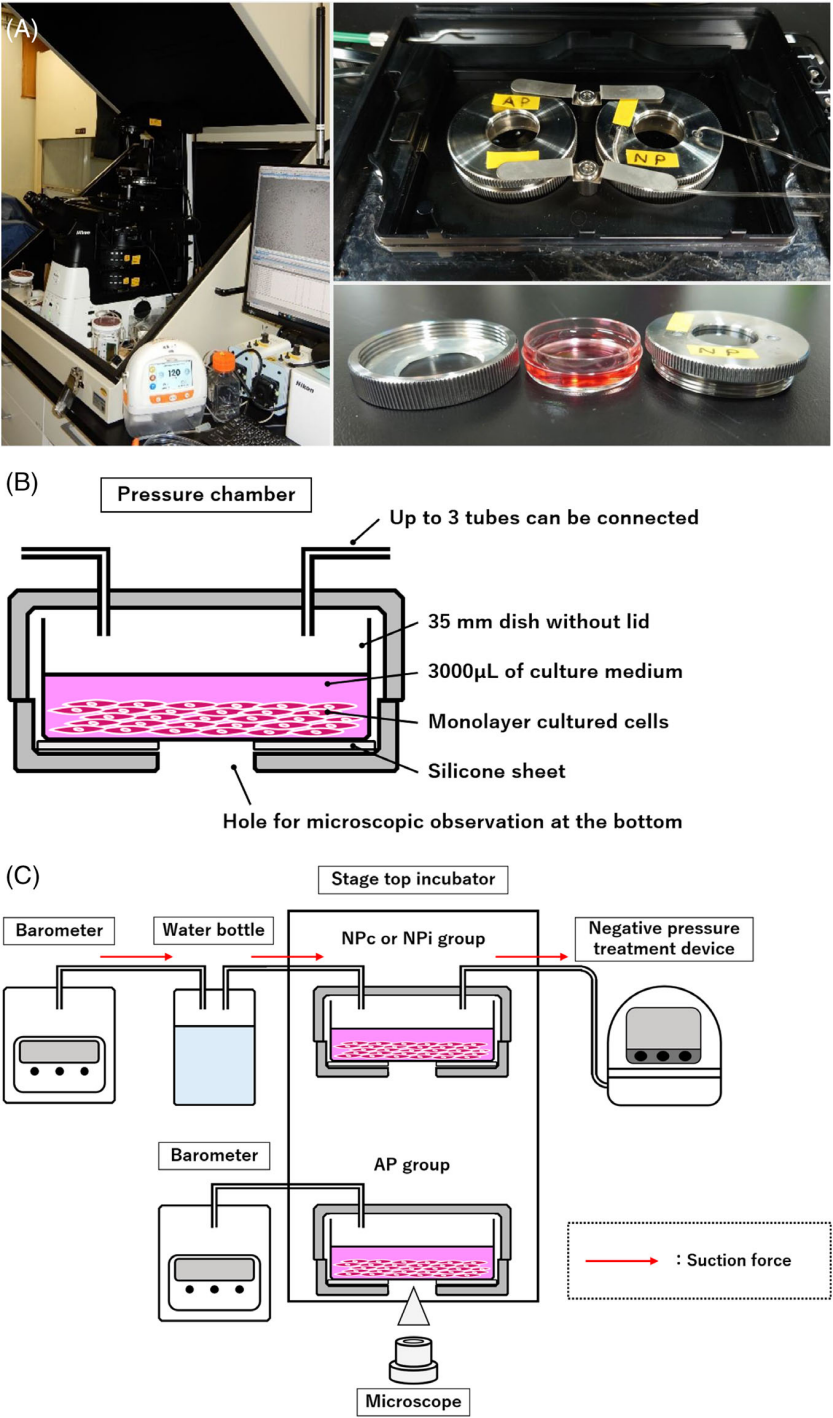
We constructed a novel negative‐pressure cell culture system by combining readily available materials. (A) Appearance of the apparatus. (Left) Overall view of the apparatus. A personal computer connected to the microscope enables cell monitoring and image analysis. (Upper right) Stage‐top incubator and two metal chambers placed there. (Lower right) The chambers just fit a 35 mm dish. (B) Schematic illustration of the sealed metal chamber for airtight culture and its internal structure. The chamber is sized to just fit a 35‐mm dish. There is a hole at the bottom for microscopic observation, and a ring‐shaped silicon sheet is placed on the bottom to ensure airtightness. The lid has three holes to which up to three dedicated connectors can be connected, enabling the exchange of gases and liquids. Cells were cultured in a monolayer using 3000 μl of filtered medium. (C) Schematic illustration of the entire apparatus. Two sealed chambers were set inside a stage‐top incubator equipped with a microscope and adjusted to ambient and negative pressure, respectively. Internal pressures were recorded by a barometer connected to the very upstream of the circuit. Negative pressure was applied using a negative‐pressure treatment device. A water bottle was placed upstream of the circuit in order to maintain high humidity during the negative‐pressure treatment, as water vapor saturation in the chamber would be disturbed and the medium would evaporate. All tubing was connected in such a way that it did not interfere with the movement of the microscope stage and cell observation.

A stage‐top temperature‐controlled incubator (Tokai Hit, Fujinomiya, Shizuoka, Japan) was installed on the stage of an inverted microscope (Nikon, Tokyo, Japan). The incubator was equipped with heaters on the floor and a lid to maintain the internal temperature at a set value. It was also equipped with a bath to maintain appropriate water vapor saturation within. A sealed metal chamber was installed in this incubator, and cells were cultured inside it (Figure 1A).

### 2.2 Negative‐pressure incubation

For negative‐pressure culture, a sealed metal chamber (Tokai Hit, Fujinomiya, Shizuoka, Japan) was used (Figure 1B). The chamber was originally designed for pressurized cell culture and fit exactly one 35‐mm dish (excluding the lid) inside; it consisted of a screw‐on lid and a pedestal section. Center of the lid was made of clear glass to let microscope light in and had three holes for connecting tubes. Up to three tubes could be connected to this chamber via a special metal connector, allowing the exchange of gases and liquids. The pedestal portion was constructed with a hole in the center slightly smaller than a 35‐mm dish for microscopic observation. A silicone rubber sheet cut to the same size as the floor of the pedestal was installed to keep the chamber airtight when the 35‐mm dish was placed on it. Two identical chambers were provided.

The two chambers were placed in the aforementioned stage‐top incubator, and cells were cultured inside. The chambers were connected to a negative‐pressure treatment device via metal connectors and silicone tube. The negative‐pressure device used was the RENASYS TOUCH (Smith & Nephew; Watford, Hertfordshire, UK), which is widely used in clinical practice. The device is capable of continuous (constant) treatment, from −25 mmHg to a maximum of −200 mmHg, as well as intermittent treatment, called AI mode, in which two set pressures are repeated at set time intervals (e.g., −120 mmHg for 5 min and −25 mmHg for 2 min in repeated cycles). The two metal chambers could be treated simultaneously, each under different pressure conditions, and the unused connector hole in the metal chamber lid was blocked with a plastic tape.

### 2.3 Cell culture procedure

Human skin keratinocyte PSVK‐1 was purchased from Japanese Collection of Research Bioresources (JCRB) Cell Bank. Cells were cultured in Keratinocyte‐SFM (Thermo Fisher Scientific; Waltham, MA, USA) supplemented with 5 ng/ml epidermal growth factor, 50 μg/ml bovine pituitary extract, and 100 μg/ml streptomycin–penicillin. Cells were passaged when they were 80%–90% confluent in T‐75 flasks. Cell detachment was done using Accutase (Innovative Cell Technologies; San Diego, CA, USA). Cells for microscopy were seeded in 35‐mm dishes and incubated under the experimental conditions described later.

### 2.4 Monitoring pressure inside the air circuit

A barometer was incorporated on top of the air circuit to monitor the internal pressure throughout the treatment (Figure 1C). The pressures of continuous negative‐pressure (NPc) and intermittent negative‐pressure (NPi) groups were recorded with and without RENASYS Softport (Smith & Nephew; Watford, Hertfordshire, UK), a uniquely constructed tubing accessory that allows the intake of tiny amounts of air without having to completely seal the air circuit. Difference in the values of NPc or NPi, and that of an ambient pressure (AP) group, under simultaneous treatment was used as the relative pressure inside the negative‐pressure device to determine whether the internal pressure was adequately maintained according to the set conditions.

### 2.5 Evaporation of culture medium

The weight of each chamber containing a dish with 3000 μl medium was recorded before and after the 24‐h experiment, and the change in weight was considered to be due to medium evaporation. From the weight change and specific gravity of the medium, medium evaporation rate of each dish per 24 h was calculated as %Medium loss, and the value was compared across the groups.

### 2.6 Scratch assay and time‐lapse imaging

Approximately 1 × 10^6^ cells of PSVK‐1 were seeded in a 35‐mm dish. After 24‐h incubation in an incubator (37°C, 5% CO_2_, and AP), the medium was changed, and cells were scratched with a 200‐μl pipette tip (Eppendorf; Hamburg, Germany). Thereafter, the cells were rinsed with another medium and replaced with 3000 μl of fresh filtered serum‐free medium. The filtration process was performed using a 0.22‐μm Durapore PVDF Membrane (Millipore Sigma; MA, USA) in order to remove large debris from the medium and obtain a clear image of cells. Cells were divided into three groups, namely, the AP, NPc, and NPi group. Each dish was allowed to settle in the custom‐made chamber fixed to the stage‐top incubator on a microscope stage and then incubated under ambient pressure (AP group), continuous −120 mmHg negative pressure (NPc group), or repetitive cycles of −120 mmHg for 5 min and −25 mmHg for 2 min (NPi group), using a previously described negative‐pressure device. Time‐lapse photography was conducted every 30 min at four different points of each dish; mean percentage of residual open wound area, compared to the initial cell‐free surface, was analyzed as the %Wound area over time using the NIS‐Elements software (Nikon; Tokyo, Japan), and the value was compared across the three groups.

### 2.7 Statistical analysis

Data were statistically analyzed using the JMP Pro 15.2.0 software (SAS Institute Japan; Tokyo, Japan). Data were statistically examined with unpaired two‐tailed *t*‐test for comparisons between two groups and one‐way ANOVA followed by the Tukey's post hoc test for comparisons across three groups. All data are presented as means and standard deviations. A *p*‐value <0.05 was considered statistically significant.

## 3 RESULTS

### 3.1 Both continuous and intermittent NPWT were successfully applied with RENASYS Softport

**FIGURE 2:**
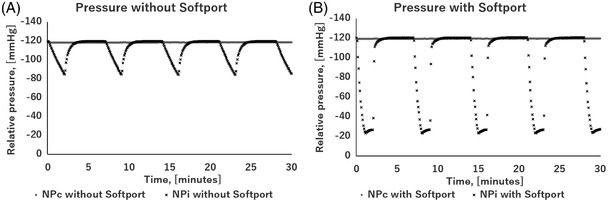
Two independent sealed culture chambers were used simultaneously in each experiment, one of which was treated under ambient pressure (AP) and the other under continuous negative‐pressure of −120 mmHg (NPc) or intermittent negative‐pressure of −120 mmHg for 5 min and −25 mmHg for 2 min (NPi). Pressure in each chamber was recorded every 5 s by a barometer, and the relative pressure in the circuit was recorded as the NPc or NPi value minus the AP value. Experiments were conducted with and without RENASYS Softport in the circuit, and results are shown in the graph. (A) Without Softport, negative pressure was maintained as set in NPc group, whereas rapid pressure increase was not obtained in NPi group, and the next high‐pressure cycle began without sufficient pressure return. (B) With Softport, negative pressure was successfully applied almost exactly as set in both the NPc and NPi groups.

When connected only with silicone tubing without the RENASYS Softport, the NPc group was able to maintain negative pressure as set; however, the NPi group could not transition smoothly from high negative pressure to low negative pressure and went back and forth between −120 and −90 mmHg compared to the −120 and −25 mmHg setting (Figure 2A).

In contrast, while using RENASYS Softport, it was possible to reproduce continuous or intermittent negative pressure as set, in both NPc and NPi groups (Figure 2B).

### 3.2 Medium evaporation rate was high in NPi group

**FIGURE 3:**
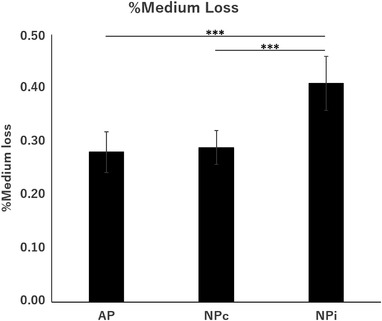
Weight of the chamber was measured before and after 24‐h treatment, and the amount of medium evaporated was calculated from the reduction and specific gravity of the culture media. The value was divided by the volume of medium at the start of the experiment (3000 μl) and defined as %Medium loss; the values were compared across different conditions. AP: ambient pressure group, *n* = 10; NPc: continuous negative‐pressure group, *n* = 5; NPi: intermittent negative‐pressure group, *n* = 5. Values are expressed as means ± standard deviation. Statistical significance is marked with ***p < 0.005.

%Medium loss was significantly higher in the NPi group (0.41% ± 0.10%) than in the other two groups (AP group, 0.28% ± 0.04%, NPc group, 0.29% ± 0.03%, *p* < 0.0001) (Figure 3). In all groups, medium evaporation had no serious effect on cell observation.

### 3.3 Negative‐pressure treatment accelerated monolayer wound healing

**FIGURE 4:**
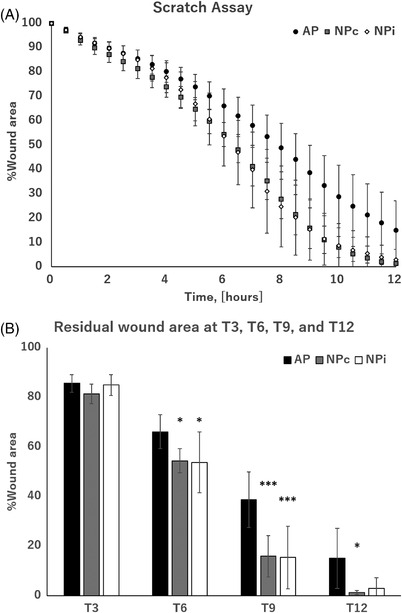
Scratch assay using the apparatus was conducted on human keratinocytes PSVK‐1. (A) Scratch wounds were created using a 200‐μl pipette tip on cells confluent in 35‐mm dishes. Cells were divided into three groups, namely, AP, NPc, and NPi, and incubated with the apparatus shown in Figure 1. Time‐lapse images of four coordinates in each dish were acquired every 30 min, and the remaining wound area relative to that in the beginning of the experiment (T0) was calculated as %Wound area. (B) The residual wound area was compared 3, 6, 9, and 12 h after the start of the experiment (T3, T6, T9, and T12). Both NPc and NPi groups had significantly less residual wound area than the AP group. AP: ambient pressure group, *n* = 10; NPc: continuous negative‐pressure group, *n* = 5; NPi: intermittent negative‐pressure group, *n* = 5. Values are expressed as means ± standard deviation. Statistical significance is marked with *p < 0.05 and ***p < 0.005.

Scratch assay using the device allowed the obtainment of temporal data on wound closure rate at specific coordinates on the dishes without ever interrupting the negative‐pressure treatment (Figure 4A). The results showed the residual wound area to be significantly smaller in the NPc (54.4% ± 4.9%, *p* < 0.05) and NPi (53.7% ± 12.3%, *p* < 0.05) groups than in the AP group (66.1% ± 6.9%) 6 h after the start of treatment (T6); the difference was at a maximum at 9 h (T9) (AP group, 38.6% ± 11.2%, NPc group, 15.9% ± 8.3%, *p* < 0.005; NPi group, 15.4% ± 12.6%, *p* < 0.005); and the difference at 12 h (T12) was smaller as wound closure was initiated in some dishes (AP group, 15.0% ± 12.1%, NPc group, 1.33% ± 0.8%, *p* < 0.05; NPi group, 2.98% ± 4.2%, *p* = 0.065) (Figure 4B). A time‐lapse video of the scratch assay for each condition is provided as Supporting Information 1.

## 4 DISCUSSION

Since the introduction of NPWT in 1997 by Morykwas et al.,^1^ its wound‐healing effects have become widely known in clinical practice. Although initially used only for open wounds, it is now widely used in wound bed preparation to achieve wound closure with skin grafts or skin flaps owing to its granulation effect.^15^ Recently, devices, such as the NPWT with instillation and dwell time (NPWTi‐d)^4^ (which repeatedly injects cleaning solution, immerses the wound, and performs aspiration), the single‐use NPWT,^16^, ^17^ (which is highly portable and causes few skin problems), and the NPWT used for temporary closure of open abdomen,^18^ have evolved significantly, owing to which, indications have expanded to include infected wounds and exposed organs, which had previously been considered unsafe.^4^ The efficacy of NPWT in preventing wound infection, primarily in closed wounds,^19^, ^20^ has been described in a Cochrane review.^20^, ^21^

Thus, a quarter of a century after the advent of NPWT, although numerous clinical studies, including RCTs^22^, ^23^ and meta‐analyses,^24^ have been reported, scientific evidence is still insufficient.^25^ This is partly because the molecular biological mechanisms of NPWT have not been fully elucidated by basic research, and the numbers of cell‐based in vitro studies are limited. One reason for the paucity of in vitro studies is that the healing mechanism of NPWT is complex and difficult to reproduce completely. Specifically, the healing mechanism is thought to be a combination of the effects of wound macrodeformation, cellular mechanical response (microdeformation), exudate drainage, and wound environment modulation by creating a closed environment, all of which interact with each other.^7^, ^8^, ^9^ Therefore, healing mechanisms should ideally be studied one factor at a time. With a better understanding of the mechanisms, decisions on dressing and equipment selection, pressure intensity and length of time, and duration of treatment, which are currently determined by the clinician's experience, could be optimized to achieve an ideal course of treatment, fewer complications, and a better wound treatment available to everyone.

Although several studies on cellular responses under negative pressure have been reported till date, there is no uniformity regarding how cells are cultured under negative pressure. Wilkes,^26^ McNulty et al.,^10^, ^27^ and Lu et al.^12^ created bioreactors that can reproduce NPWT using foam dressing on 3D cultured cells; Hsu et al.^28^, ^29^, ^30^, ^31^ cultured cells in an incubator under negative pressure and evaluated cell morphology and proliferation in real time during negative‐pressure treatment using the electrical cell‐substrate impedance sensing system, which detects and evaluates the electrical impedance of cells and the cell substrate. Shou et al.^13^ directly aspirated flasks, and Pandit et al.^14^ used Ziploc bags to create a negative‐pressure environment. Overall, previous studies have varied from custom‐built incubation devices to simple methods of creating a negative‐pressure environment; however, the former has challenges in terms of limited laboratories where experiments can be performed and reproducibility, whereas the latter requires sequential interruptions of negative‐pressure treatment for the observation of cells and does not allow real‐time morphological observation. We aimed to develop a new culture system that could circumvent these problems, a device that is simple to construct and reproducible, and the one that allows real‐time monitoring of cells using a microscope without interrupting negative‐pressure treatment (Figure 1A,B). Although intermittent negative pressure has recently been reported to be more effective than continuous negative pressure for cure of wounds,^6^ there has been no cell‐based experiment yet that could verify this fact. Using the negative‐pressure treatment device RENASYS (widely used in clinical practice) as the treatment source, our novel culture system was able to reproduce the treatment conditions actually used for patients in both continuous and NPi modes.

Pressure monitoring using a barometer was performed by this device. Results revealed that even if the NPWT device did not sound an alarm and appeared to be working properly, pressure in the chamber was sometimes not of the intended values (Figure 2A). It is known that the pressure exerted on the wound surface during NPWT does not always match the machine settings.^32^ In in vitro experiments, ambiguity in parameter settings can cause uncertainty of results and confusion among researchers. Mechanical stimulation of the wound through cycles of high negative pressure and low or no negative pressure is thought to enhance wound‐healing effects, such as stimulation of angiogenesis.^5^ Therefore, we found that if the device is too airtight, recovery from high negative pressure may not be smooth, and the intended therapeutic effect may not be achieved (Figure 2A). To address this problem, we used RENASYS Softport, a standard accessory for RENASYS, which has an aeration disc in the middle of the tubing to allow microscopic gas exchange without complete closure of the circuit. This would allow rapid recovery from high negative pressure to low negative pressure during intermittent treatment, thereby reproducing the treatment cycle as set by the apparatus (Figure 2B).

When negative‐pressure treatment was performed under conditions of constant micro gas exchange, medium evaporation from the culture dish became a major problem. Depending on the conditions, all the initial culture medium (3000 μl) evaporated in a few hours; however, this problem could be avoided by installing a water bottle in the circuit. Although the %Medium loss was significantly higher in the NPi group than in the AP and NPc groups, it was still low at 0.41% at 24 h and did not have significant effect on cell observation (Figure 3).

Due to the nature of cell culture on the stage of an inverted microscope, the system allowed imaging without interrupting the negative‐pressure treatment of cells, and almost all functions of the microscope software could be used. As the entire process of moving the stage to the specified coordinates, image acquisition, and image analysis can be performed by software, it is believed that technical bias can be minimized. Scratch assay using human keratinocytes, based on the obtained time‐lapse images, revealed that cell migration was significantly enhanced in the NPc and NPi groups, compared to that in the AP group (Figure 4A,B). Previous reports have indicated that continuous negative pressure^31^ or microgravity^33^ promotes epithelial‐to‐mesenchymal transition (EMT), in which the epithelial nature of keratinocytes is weakened and the mesenchymal nature is strengthened,^34^ resulting in weakened cell‐substrate adhesion and enhanced cell migration. However, the results of our experiments suggested that a similar phenomenon may occur, not only under continuous negative pressure, but also under intermittent negative pressure. Additionally, the results supported the culture method using this apparatus to be comparable to the conventional negative‐pressure culture method. Our apparatus did not require complicated equipment and could easily be constructed by combining readily available materials.

This study focused on microdeformation as part of the major therapeutic mechanisms of NPWT. In NPWT, a sudden pressure difference between inside and outside the dressing occurs as soon as treatment begins, resulting in a compression force on the wound surface^32^ and hypoxia in the dressing.^35^ This rapid and transient environmental change is thought to induce cellular responses such as the release of angiogenetic factors.^36^ Although the commonly used condition of −120 mmHg negative pressure corresponds to a difference in altitude of approximately 1500 m, it is thought that wound healing is rather delayed in high‐altitude residents due to tissue hypoxia.^37^, ^38^, ^39^ For these reasons, we believe that negative pressure and low pressure should be considered separately, although this distinction has not been made in the past, and care should be taken to set up experimental systems, rather than simply culturing cells at high altitude.

Limitations of this apparatus include the following: Because the culture medium perfusion system is not yet complete, microscopic observation and image analysis may be disturbed by floating debris, such as dead cells, during incubation periods longer than 24 h. Because only two 35‐mm dishes, at most, can be observed simultaneously, it is not possible to compare multiple parameters; thus, the number of trials should be increased. As a wide variety of cells exist in an actual wound site and interact with each other during wound healing, future experiments using cocultures of multiple cell types will also be necessary. Here, we focused on epithelial cells, which have received minimal attention in context of NPWT. As NPWT has traditionally been used for skin defect wounds, epithelial cells have not been present in the treated site except at the wound edge. It is known that hypoxia at the wound margin caused by microdeformation promotes angiogenesis via the hypoxia‐inducible factor‐1α (HIF‐1α) to vascular endothelial growth factor pathway.^40^ As keratinocytes in psoriasis patients are known to be an important source of HIF‐1α and contribute to skin angiogenesis,^41^, ^42^, ^43^, ^44^ we believe the significance to study the role of keratinocytes, including angiogenesis, in the mechanisms of NPWT. Recently, NPWT for primary closure, which is called incisional NPWT (iNPWT), has attracted attention^19^ and is used as an alternative to postoperative dressings, so there is epithelium at the site of the treatment. Although iNPWT has been shown to reduce surgical site infection, it has been linked to an increase in skin disorders such as blistering.^21^ Patient genetic profiling studies suggest that NPWT may initially enhance reepithelialization by promoting epithelial cell migration and proliferation but may inhibit epidermal maturation in the long term.^45^ We will continue to study how keratinocytes contribute to NPWT‐induced angiogenesis and the possibility that EMT of epithelial cells induced by NPWT may contribute to the reepithelialization of wounds and be one of the causes of iNPWT‐induced skin problems. As for future improvements, variations of intermittent therapy, such as cyclic NPWT,^46^ can be reproduced using a V.A.C. Therapy System (3 M, Saint Paul, MN, US) or other equipment, instead of RENASYS, as the negative‐pressure source. The apparatus could also be used to acquire fluorescent images, and with further modification, it could reproduce long‐term cultures and NPWTi‐d by perfusion of culture medium using the three ports of the metal chamber. We hope that our apparatus will accelerate in vitro studies on NPWT and provide high‐quality molecular biological evidence regarding its therapeutic effects.

## 5 CONCLUSION

We successfully developed a negative‐pressure cell culture system, that is, capable of monitoring cells and acquiring images in real time while performing NPWT, including continuous and intermittent treatments, on cells. Although further improvements would be required, experiments using this device may be expected to provide high‐quality molecular biological evidence regarding NPWT.

## CONFLICT OF INTEREST

The authors declare no conflict of interest.

## Supporting information

Supporting information 1 Time‐lapse video of the scratch assay. Images were acquired every 30 min for 24 h from scratch wound creation. Representative examples are shown that are close to the average of each group. Scale bar indicates 500 μm. AP, ambient pressure group; NPc, continuous negative‐pressure group; NPi, intermittent negative‐pressure group.

## Data Availability

The datasets generated during and/or analyzed during the current study are available from the corresponding author on reasonable request.
